# Microstructure and Properties of Porous High-N Ni-Free Austenitic Stainless Steel Fabricated by Powder Metallurgical Route

**DOI:** 10.3390/ma11071058

**Published:** 2018-06-22

**Authors:** Ling Hu, Tungwai Ngai, Hanlin Peng, Liejun Li, Feng Zhou, Zhengwu Peng

**Affiliations:** 1 National Engineering Research Center of Near-Net-Shape Forming Technology for Metallic Materials, South China University of Technology, Guangzhou 510640, China; 201510100536@mail.scut.edu.cn (L.H.); dhni@scut.edu.cn (T.N.); zhoufeng@scut.edu.cn (F.Z.); pengzw@scut.edu.cn (Z.P.); 2 State Key Laboratory of Material Processing and Die & Mould Technology, Huazhong University of Science and Technology, Wuhan 430074, China; penghl@hust.edu.cn

**Keywords:** porous morphology, microstructure, nitrides, crystallographic feature, elastic modulus

## Abstract

Porous high-N Ni-free austenitic stainless steel was fabricated by a powder metallurgical route. The microstructure and properties of the prepared porous austenitic stainless steel were studied. Results reveal that the duplex stainless steel transforms into austenitic stainless steel after nitridation sintering for 2 h. The prepared high-N stainless steel consists of γ-Fe matrix and FCC structured CrN. Worm-shaped and granular-shaped CrN precipitates were observed in the prepared materials. The orientation relationship between CrN and austenite matrix is [011]_CrN_//[011]_γ_ and (-1-11)_CrN_//(1-11)_γ_. Results show that the as-fabricated porous high-nitrogen austenitic stainless steel features a higher mechanical property than common stainless steel foam. Both compressive strength and Young’s modulus decrease with an increase in porosity. The 3D morphology of the prepared porous materials presents good pore connectivity. The prepared porous high-N Ni-free austenitic stainless steel has superior pore connectivity, a good combination of compressive strength and ductility, and low elastic modulus, which makes this porous high-N Ni-free austenitic stainless steel very attractive for metal foam applications.

## 1. Introduction

Metal foams have attracted much attention due to their unique combination of properties: high specific strength, low density, low elastic modulus, lightweight and high energy absorption capacity [1]. They can be used in numerous applications, such as lightweight constituents, filters, biomedical implant materials, vibration dampers and heat exchangers [2]. In porous materials, the mechanical properties, including density, compressive strength, and elastic modulus, can be adjusted by controlling the preparation parameters and the porosity [3,4]. Stainless steel foams have been made through several methods, and the microstructure, mechanical properties and corrosion resistance have been researched [1,2,5,6,7], showing good potential to be a good alternative for metal foams in applications that require lightweight, excellent mechanical properties and good corrosion resistance.

High-N austenitic stainless steels (HNASS) have attracted great interest due to their excellent mechanical properties, high N content and absence of the Ni element, compared with common austenitic stainless steels [8,9,10]. High-N austenitic stainless steel has been used for implant devices by Yang et al. [11] and Li et al. [12], for example as coronary stent material, showing superior strength and better hem-compatibility. The idea of fabricating HNASS foam could also open up new potential applications in the metal foam area. For N-containing steel, N is not only used to substitute Ni to avoid the allergy problems caused by Ni, but also to improve the properties of the steel [13,14,15]. Alvarez et al. [16] fabricated and studied Lotus-type porous Ni-free stainless steel, showing great promise for medical implant applications, also demonstrating anisotropy in its compressive property and elastic modulus. Porous Ni-free stainless steel fabricated by powder metallurgy (PM) has the characteristic of isotropy in porosity, pore attribution and properties. Therefore, fabrication methods of porous HNASS are mostly based on the powder metallurgical route. Garcia-Cabezon et al. [7] characterized porous N-containing austenitic stainless steel, which is very suitable for biological applications. However, there is little reported about the effect of fabrication parameters on the microstructure and mechanical strength of porous HNASSs. Thus, a systematic study on the fabrication, microstructure and mechanical properties of HNASSs is necessary for further promotion of the application of HNASS foam.

Alloying steels with N is not an easy task. Uggowitzer et al. [9], Vanderschaeve et al. [17] and Simmons [18] studied conventional high-N austenitic stainless steels obtained by high-pressure melting techniques under high N pressure, which requires expensive equipment. Compared with casting methods, high-N stainless steel produced by powder metallurgy does not require complicated and expensive equipment to obtain high N content [19,20] and has the characteristic of controlled porosity and pore features [3], without segregation. For PM method, N_2_ is widely used as a protective atmosphere during sintering [21,22]. The gas-solid interaction between N_2_ and stainless steel during the nitriding and sintering process increases the N content and contributes to the phase transformation from ferrite to austenite [23].

It is important to note that despite several published studies on porous stainless steel prepared by casting methods or the powder metallurgical method, porous high-N Ni-free austenitic stainless steels have rarely been studied. In this study, porous austenitic stainless steels with high N content (more than 3 wt. %) were fabricated via the gas-solid nitriding and sintering process. The microstructure, phase transformation and mechanical properties of the porous high-N Ni-free austenitic stainless steels were systematically investigated to evaluate the possibility of its being considered as high strength metal foam.

## 2. Experimental

**Figure 1:**
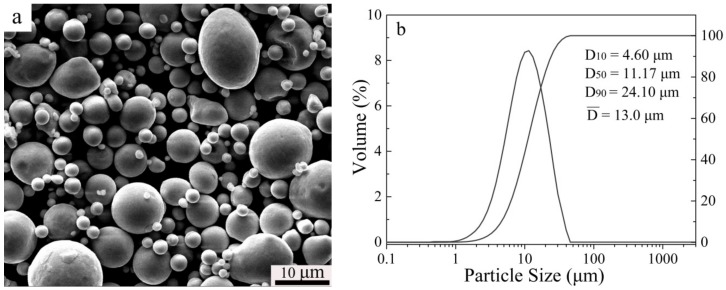
SEM morphology (**a**) and particle size distribution (**b**) of the as-received powders.

Spherical N_2_ gas atomized duplex stainless steel powders with an average particle size of 13 μm were used, with chemical compositions of 17.75 Cr, 10.84 Mn, 3.40 Mo, 0.36 Ni, 0.059 C, 0.44 N, 0.134 O (wt. %), and balanced with Fe. The morphology and particle size distribution are shown in Figure 1. The theoretical density of the as-received powders is 7.69 g/cm^3^. Their chemical composition was provided by the supplier, Advanced Technology & Materials Co., Beijing, China. The pore former consisted of ammonium bicarbonate particles (ABP) and polyvinyl alcohol (PVA), which have been widely used in earlier research [2]. ABP (average 75 μm) was used as the space holder with the amounts 0 wt. %, 10 wt. %, 20 wt. %, 30 wt. %. Polyvinyl alcohol solution (5 wt. % PVA and 95 wt. % water) was adopted as binder to enhance strength of the green compacts. The as-received powders and space holder were mixed in a V-type mixing machine for 24 h. A cold forming process was carried on a hydraulic press machine at a pressure of 374 MPa. Pre-sintering was carried out at 200 °C for 1 h and 400 °C for 1 h to eliminate the pore former. The sintering process (at 1120 °C, 1200 °C and 1250 °C for 2 h under a flowing N_2_ atmosphere, 1 atmosphere pressure) was carried out in a tube furnace with a slow heating rate and cooling rate of 5 °C/min. All sintered samples were cooled with the furnace. All samples were subjected to solution treatment at 1150 °C for 60 min in argon atmosphere and followed by water quenching. Table 1 shows the sample codes and corresponding detailed processing parameters.

The porosity of the as-fabricated porous alloys was investigated through the Archimedes method after filling the pores with a melted wax. The N content in bulk alloys was measured by oxygen and nitrogen gas analyzer (TC600, LECO, St. Joseph, MI, USA). The microstructure of the sintered alloys was observed by scanning electron microscope (SEM, ZEISS, Merlin, Germany) without etching. Phase constituents were examined by X-ray diffraction (XRD, D/MAX-2500/PC; Rigaku Corp., Tokyo, Japan) with Mo Kα radiation. To compare it with the published literature, the XRD data was converted from Mo Kα radiation patterns to Cu Kα radiation patterns. Further phase identification was carried out by transmission electron microscope (TEM, Tecnai G2 F20 S-TWIN, FEI, Hillsboro, OR, USA). Samples for TEM analysis (3 mm in diameter) were prepared by grinding the samples to 45 μm thick and then followed by argon ion beam thinning. X-ray 3D microscope (nanoVoxel-2702, Sanying Precision Instruments Co., Ltd., Tianjin, China) was used to examine the 3D morphology of the as-fabricated porous materials. The compressive mechanical properties of the sintered samples, with a dimension of 2 mm in diameter and 4 mm in height, were examined by using a universal testing machine (MTS Test Star 810, MTS, Eden Prairie, MN, USA) at a strain rate of 1 × 10^−3^ s^−1^. Compressive yield stress (σ_0.2_) of all the samples was determined by a 0.2% deviation from the linear elastic stage of the true stress-strain curves in a normal compression test. The elastic modulus of the sintered porous alloys was measured using an in situ nanoindentation tester (TI750, Hysitron, Minneapolis, MN, USA) with a 5 mN force.

## 3. Results

### 3.1. Microstructure Characterization

**Figure 2:**
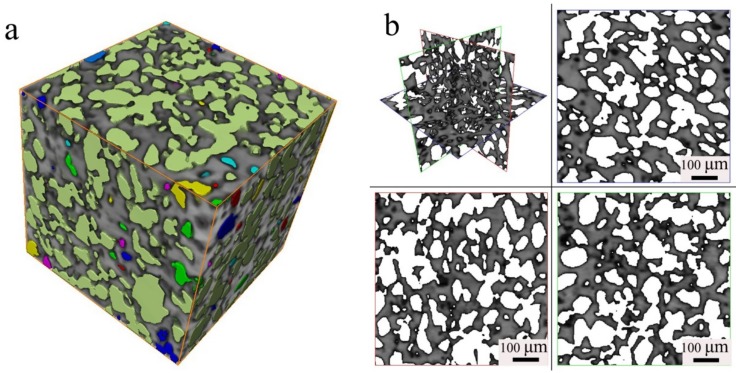
3D Micrograph of the N1120-30-374 alloy: (**a**) Three-dimensional pore distribution (**b**) four views of pore distribution.

Figure 2 shows the microstructure in three dimensions for the sintered porous material A1 (N1120-30-374). The inner porous morphology can be built up layer by layer using X-ray 3D diffraction with a high resolution of 2 μm. Figure 2a presents the morphology of the sample measured with the size of 560 μm × 560 μm × 560 μm. Gray areas are metals and colored areas are holes with various porous size and diameter. Porosity can also be determined accurately in this method. The porosity of the tested sample was determined to be 42%, lower than the result of 51.8% by the Archimedes method. There are both large amount of interconnected porous in three-dimensional space, as shown in Figure 2b. According to the research by Li et al. [4], such microstructures will promote the biocompatibility of the potential porous biomaterials. An interconnected porous structure is beneficial for cell ingrowths and body fluid transport [4]. Porous structure prepared by powder metallurgical methods is also good for reducing elastic modulus, thus reducing mismatch between implant and bone tissue.

**Figure 3:**
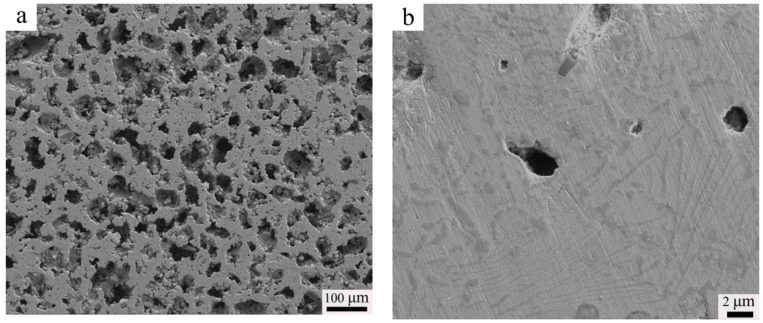
SEM-SE images of the as-sintered alloy A_1_ (**a**) low magnification; (**b**) high magnification.

**Figure 4:**
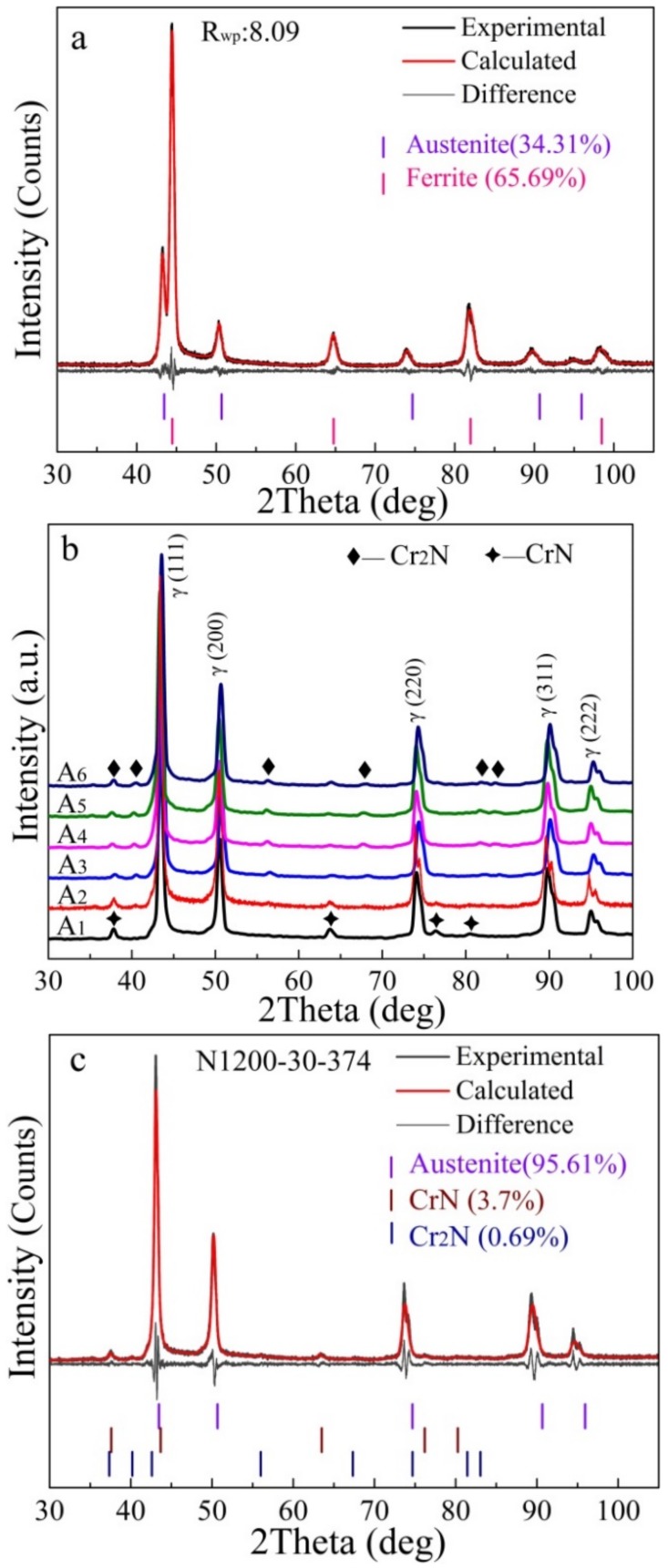

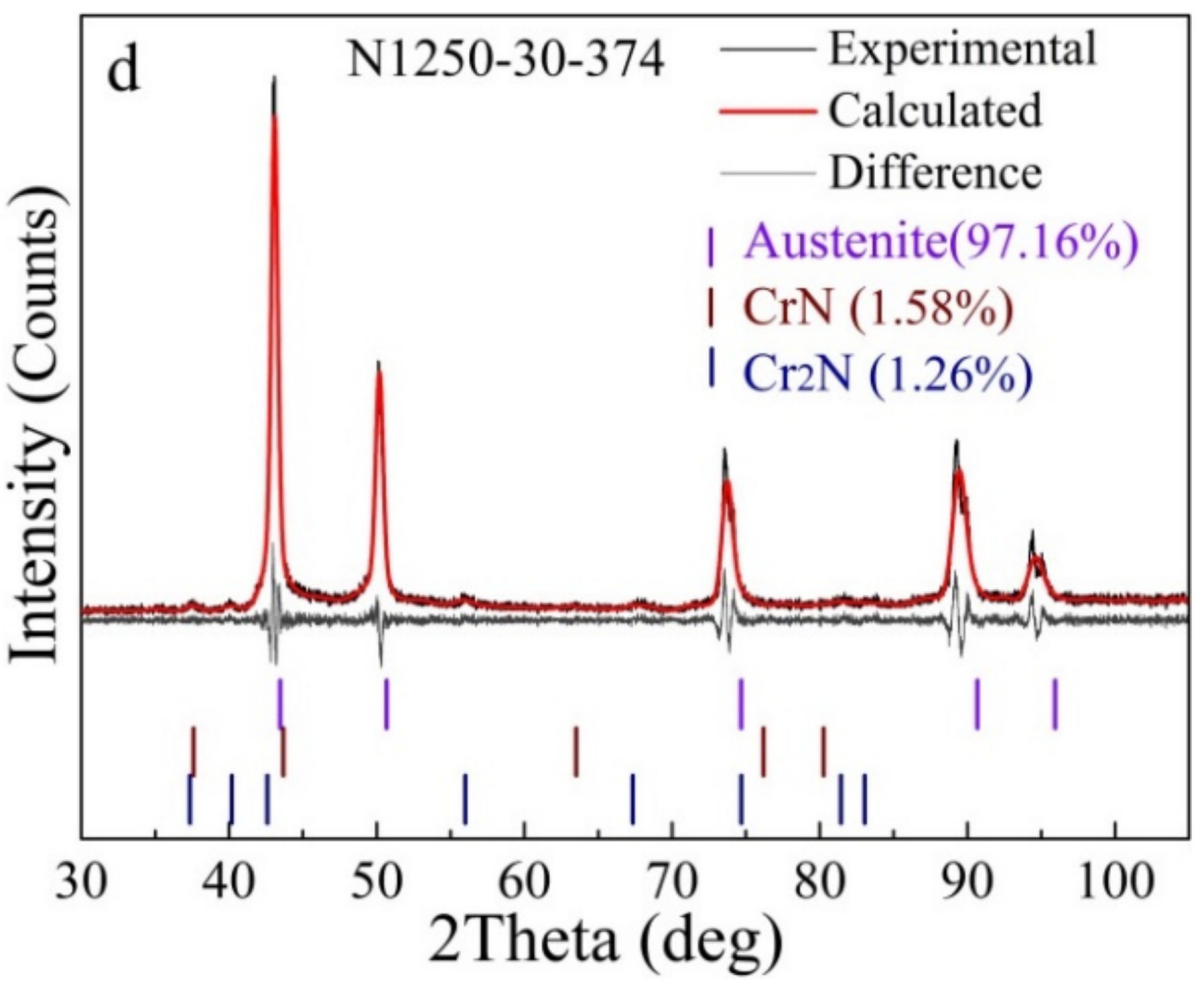
Refined XRD patterns of alloys: (**a**) the as-received powders; (**b**) XRD patterns of the as-fabricated alloys; refined XRD patterns of alloys: (**c**) N1200-30-374; (**d**) N1250-30-374.

Figure 3 presents the SEM-SE image of the sintered alloy. After sintering at 1200 °C for 2 h, the porosity of high-N austenitic stainless steel (N1200-30-374) reached 45.0%, and the corresponding morphology of pores is shown in Figure 3a. The Macropores in the microstructure formed by the elimination of the space holder and the micropores formed due to incomplete sintering of the powders. For the sintered porous alloys, a large amount of worm-shaped and granular-shaped chromium nitrides were observed, as shown in Figure 3b. This is in agreement with previous work suggesting that Cr-rich precipitates (CrN or Cr_2_N) in stainless steels may have different morphologies [24,25]. Pettersson et al. [26] studied the precipitation of chromium nitrides for the DSS 2507 with 0.27–0.28 wt. % N and found that CrN and Cr_2_N were both rational to form in the steel, considering the driving force for precipitation. According to [27,28,29], Cr2N is the precipitate commonly found in N containing duplex stainless steel (DSS) with a 0.19–0.3 wt. % N content, but CrN is seldom seen and analyzed. In the investigation of high-N austenitic stainless steels with 0.69 wt. % N [18], only hexagonal-type nitride, Cr_2_N, was observed. From the XRD patterns in Figure 4, the obvious presence of the CrN diffraction peak confirmed the precipitation of CrN phase. The Cr_2_N diffraction peak was unobvious merely from the XRD pattern, indicating that the amount of Cr_2_N was small, or maybe below the detection limit.

### 3.2. Microstructural Evolution

Figure 4 displays XRD patterns of the as-received powders and the sintered porous alloys fabricated by gas-solid nitriding and sintering. The as-received powders were found to be composed of austenite and ferrite. Phase quantification based on the XRD pattern using the whole pattern fitting method by TOPAS software (version 4.2, Bruker, Karlsruhe, Germany) was conducted and showed that the as-received material consisted of 34.31 wt. % austenite (space group Fm-3m) and 65.69 wt. % ferrite (space group Im-3m), as shown in Figure 4a. Meanwhile, diffraction peaks of CrN phase were detected in addition to the diffraction peaks of austenite, but none of the ferrite peaks could be detected in the sintered porous alloys, as shown in Figure 4b. It can be seen that the matrix of the sintered porous alloys is austenite, and small amounts of chromium nitrides were also formed. Phase quantification results show that N1200-30-374 alloy consists of 3.7% CrN and 0.69% Cr_2_N, and N1250-30-374 alloy consists of 1.58% CrN and 1.26% Cr_2_N, respectively. The N content in the alloys N1120-30-374, N1200-30-374, N1250-30-374 were examined to be 3.38, 3.34 and 3.24 wt. %, respectively.

It can be concluded that the type and relative content of the precipitate were determined by the N content. The precipitate sequences of Cr nitrides in N-containing stainless steel are Cr_2_N, Cr_2_N + CrN, CrN with increasing N content. There was a slight decrease in CrN content and a slight increase in Cr_2_N content as the nitriding and sintering temperature rose from 1200 °C to 1250 °C. The aforementioned phenomenon can be attributed to the minor decrease in N content from 3.34 wt. % to 3.24 wt. %. Xu et al. [30] also observed similar results, which result from the faster movement of atoms at higher temperature, and thus the instability of the lattice leads to some N atoms being released from the steel. Apart from the nitrides, a large amount of N existed in solution state, forming interstitial solid solutions, enlarging the austenite phase region and stabilizing austenite phase. The duplex stainless steel transformed to austenitic stainless steel after nitriding and sintering. This is consistent with the reported work that N content reached 1 wt. % in a Fe-23Cr alloy and thus the N absorption caused a structure change of matrix from ferrite to austenite [23].

**Figure 5:**
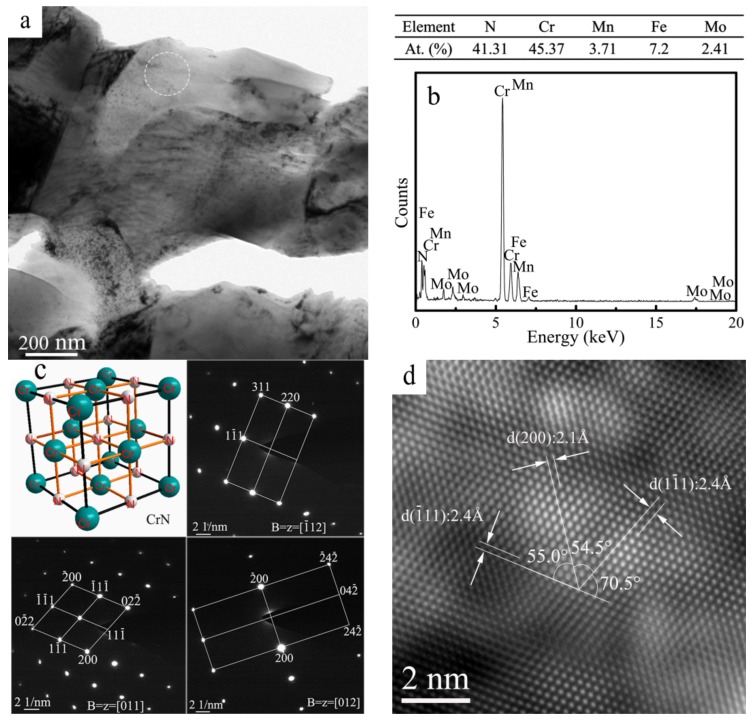
(**a**) TEM micrograph of the N1120-30-374 alloy; (**b**) EDS result of the precipitate; (**c**) Corresponding SAED patterns; (**d**) Corresponding HRTEM image of the CrN.

In N-containing stainless steels, the specific type of chromium nitrides usually depends on N content, and CrN tends to form in high-N steels. As is widely known, the presence of CrN and Cr_2_N usually occurs in nitrided stainless steels [31], but it is not an easy task to distinguish one type of nitride from another only by its morphologies [32]. Figure 5a presents the TEM micrograph of the N1120-30-374 alloy. Quantitative TEM-EDS point analysis shows that the granular-shaped precipitate found in Figure 5a is CrN, with a composition of 45.37 at. % Cr and 41.31 at. % N, as shown in Figure 5b. In order to determine and confirm the chromium-rich phase, corresponding three tilted selected area electron diffraction (SAED) patterns (B = [011], B = [01], B = [−112]) were obtained, as shown in Figure 5c. Examination of SAED patterns at three orientations suggests that the analyzed phase had an FCC structure. A high-resolution transmission electron microscope (HRTEM) image was obtained to identify the detailed lattice parameter, as shown in Figure 5d. The lattice constant of the precipitate marked in Figure 5a is 0.415 nm. Therefore, analysis results of chemical composition, crystal structure and lattice parameter suggest that the precipitate was CrN compound. A previous study, [26], concluded that the formation of the meta-stable CrN was induced by higher cooling rates with 0.27–0.28 wt. % N content in a DSS. However, due to the high N content and the long sintering and cooling time, the CrN phase in this study was precipitated and existed in the as-fabricated high-N austenitic stainless steel.

**Figure 6:**
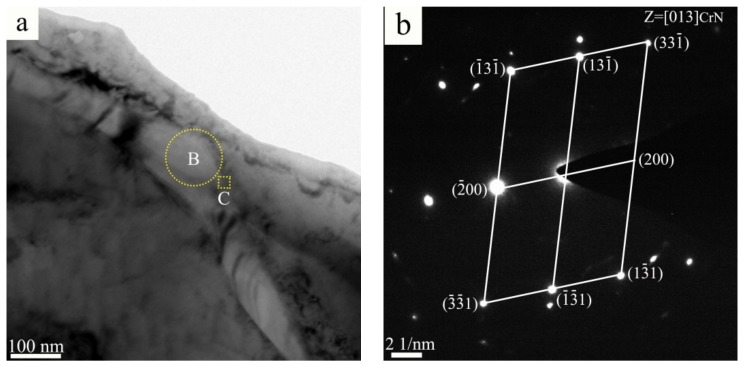

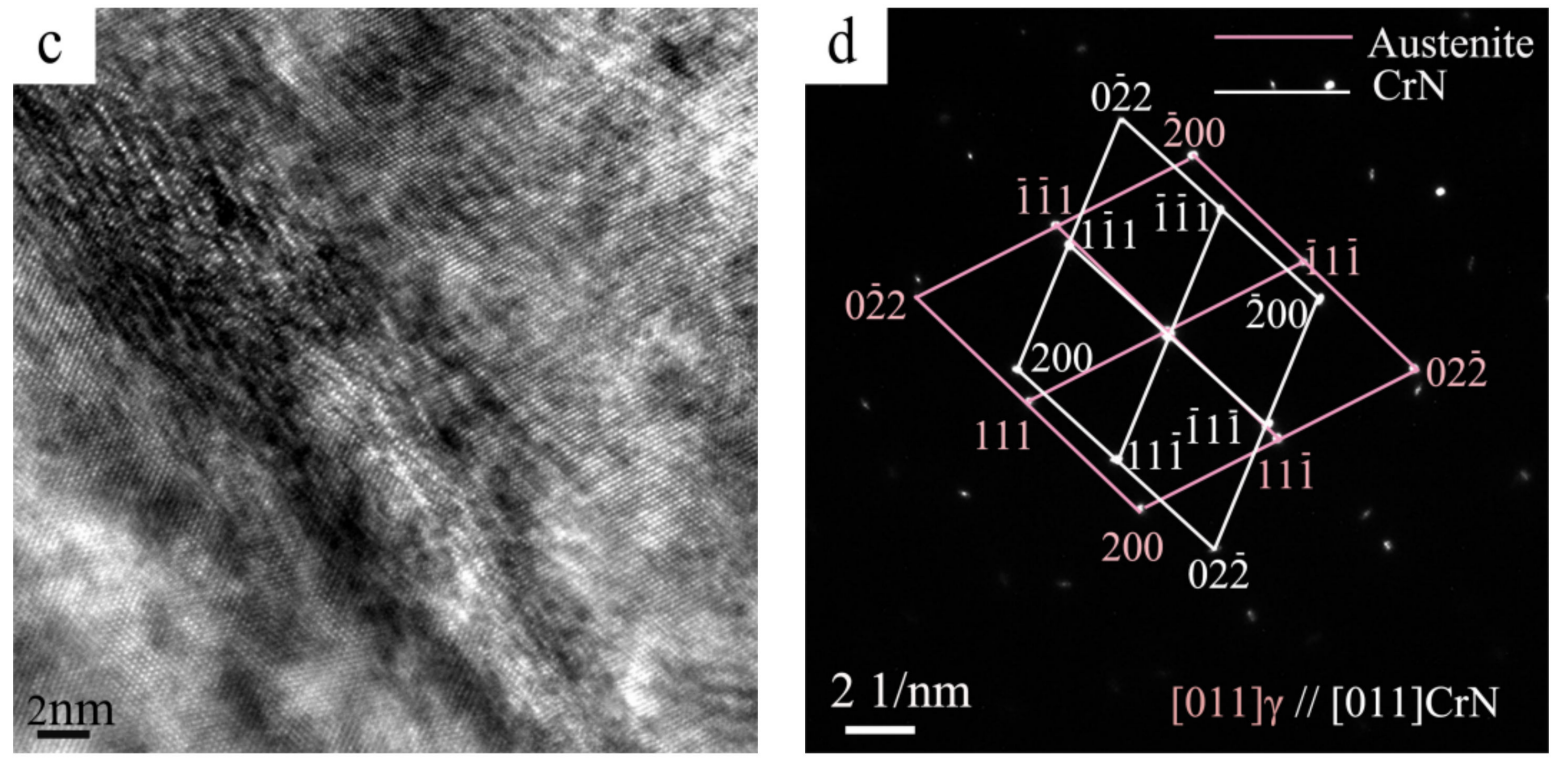
(**a**) TEM micrograph of the worm-shaped precipitate in N1120-30-374 alloy; (**b**) The corresponding SAED patterns of the worm-shaped precipitate; (**c**) HRTEM of interface between the worm-shaped CrN phase and γ; and (**d**) corresponding FFT patterns of Figure 6c.

A typical TEM micrograph of the worm-shaped precipitate in the sintered alloy is shown in Figure 6a. Quantitative TEM-EDS point analysis results show that the worm-shaped precipitate is rich in Cr and N. The corresponding SAED pattern in Figure 6b confirms that the structure of the worm-shaped precipitate is FCC, indicating that the worm-shaped precipitate is CrN phase. It can be concluded that both the worm-shaped precipitate and the granular-shaped precipitate are CrN phase, only with different morphologies, in the N1120-30-374 alloy with 3.38 wt. % N. Figure 6c is the HRTEM image of the phase interface between the worm-shaped CrN and γ-Fe matrix. The interface has a width of around 5 nm and the misfit of the boundary is observed. The Fast Fourier transformed (FFT) pattern shows that the orientation relationship between CrN and austenite matrix is [011]_CrN_//[011]_γ_ and (-1-11)_CrN_//(1-11)_γ_, with around 1.5 misorientation, as shown in Figure 6d. Due to the small amount or absence of Cr_2_N in the N1120-30-374 alloy, Cr_2_N phase was not observed in the TEM test.

### 3.3. Mechanical Properties

**Figure 7:**
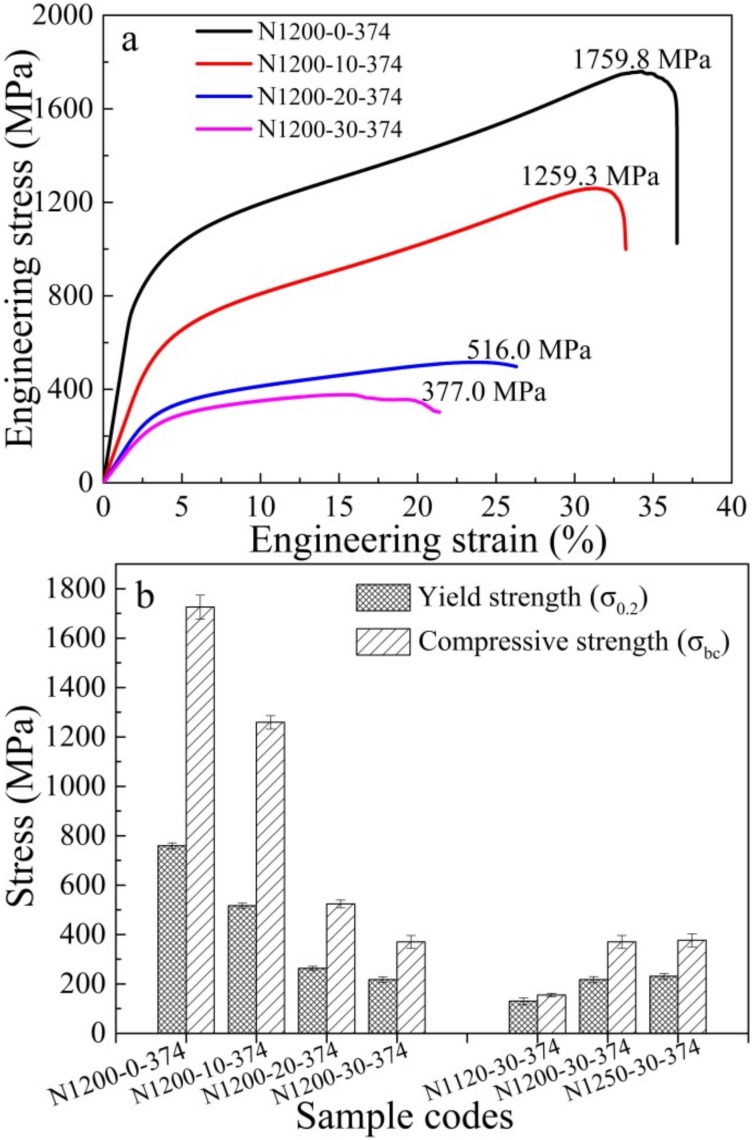
Engineering stress-strain curves for the porous HNASSs with various amounts of space holder (**a**); Yield strength and compressive strength of the porous HNASSs with various sintering temperatures and amounts of space holder (**b**).

Figure 7a shows room-temperature compressive stress-strain curves for porous HNASSs with different amounts of space holder. It is worth noticing that all the alloys have similar stress-strain curves. All curves increase linearly in the initial stage, corresponding to the linear elastic region of the typical engineering stress-strain curves. The average compressive strength of the porous HNASSs reduced from 1259.33 MPa to 377.0 MPa with the increase of space holder from 10 to 30 wt. %. The as-sintered porous HNASSs present a large fracture strain of about 25–30%, showing good ductility, which is dramatically higher than the engineering strain of biomedical porous Ti alloys prepared by the PM method [4]. The better ductility of the as-sintered materials can be ascribed to the FCC structured austenitic matrix. Figure 7b shows the variation of compressive strength and yield strength of the as-fabricated porous materials with various amounts of space holder and sintering temperature. The variation trends of yield strength and compressive strength are similar to one another. As the sintering temperature rises from 1120 °C to 1250 °C, the compressive strength and yield strength increases from 155.1 MPa and 129.9 MPa to 383.2 MPa and 224.8 MPa.

There is a big difference in the compressive stress-strain curves between the as-fabricated HNASS foams and other metallic foam [33,34,35,36], which commonly consist of a linear elasticity region, an elastic-plastic transition zone, a plateau region and a densification region. There are only linear elastic, elastic-plastic transition and plateau regions, but none of the obvious densification region after the plateau region in the stress-strain curves. The stress-strain curves decrease abruptly, which denotes a rupture, in the last period during the compression tests after a strain of 30–35%. This phenomenon may be ascribed to the severe interaction effect of dislocations and the second phase (especially the nitrides) in the high-N stainless steel. A mass of dislocations is formed, gliding and then tangling around the nitrides accompanied by plastic deformation. Numerous dislocations cause concentration of stress at the phase boundary of nitrides and matrix, and finally crack formation [37]. Crack formation and growth cause the rupture of the compression tests.

Table 2 shows the porosity, elastic modulus, compressive strength and yield strength of the porous high N austenitic stainless steels. Obviously, the porosity of the materials increased and the properties decreased gradually with the increasing amount of space holder. The porosity of the sintered porous samples increases from 14.3% to 45.0% with increasing amount of space holder from 0 to 30 wt. % sintered at 1200 °C for 2 h. The porosity of the materials with the same NH_4_HCO_3_ content decreased from 51.8% to 42.6% when increasing the sintering temperature from 1120 °C to 1250 °C. The number of micropores decreased with an increase in sintering temperature, leading to a slight decrease in porosity. There is no doubt that higher sintering temperature results in densification of the whole structure. Therefore, the porosity decreases to 42.6% and the compressive strength increases to 383 MPa when sintering temperature rises to 1250 °C.

**Figure 8:**
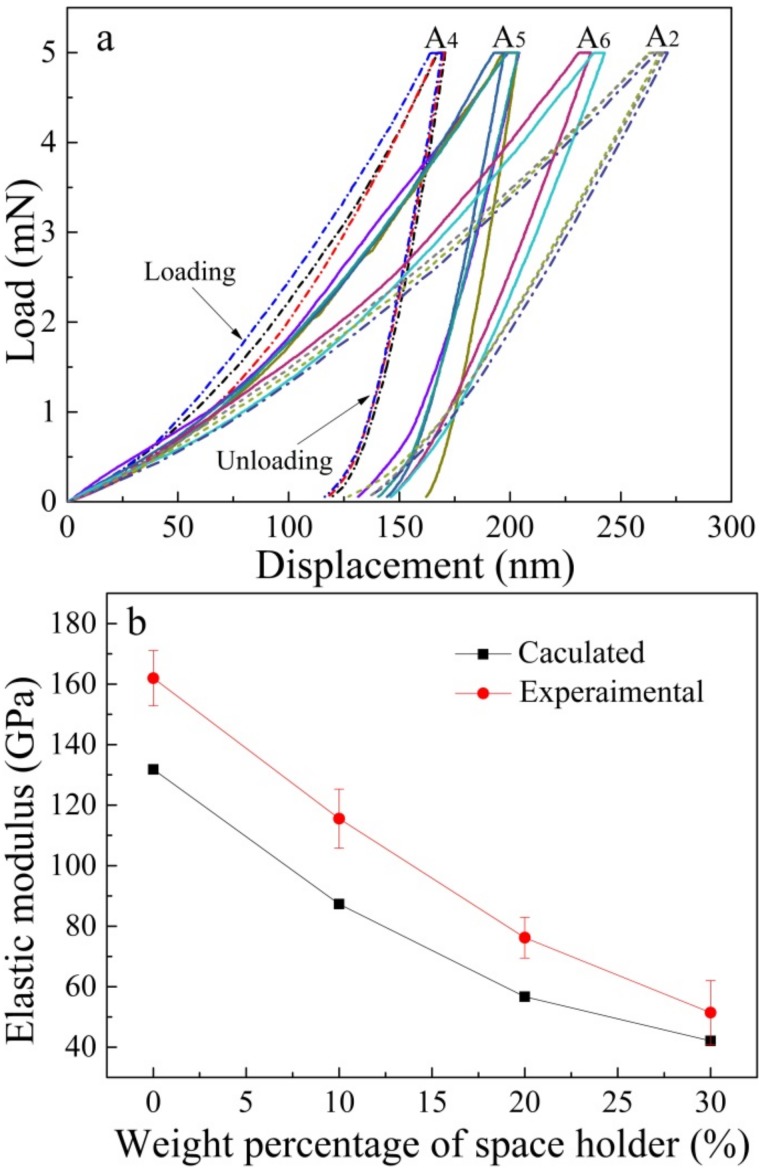
Selected load-displacement curves of the porous HNASSs with different amounts of space holder (0 wt. %, 10 wt. %, 20 wt. % and 30 wt. %) sintered at 1200 °C for 2 h (**a**); Experimental and calculated elastic modulus (**b**).

Figure 8a shows the load-displacement curves of the sintered porous materials for the nanoindentation tests. Several curves of each sample are presented. As shown in Figure 8a, the average penetration depth of samples with 0 wt. %, 10 wt. %, 20 wt. % and 30 wt. % space holder were 150 nm, 175 nm, 225 nm and 270 nm, respectively, with a maximum load of 5 mN. The penetration depth of each sample fluctuated in a small range, showing excellent repeatability of the elastic modulus for the as-sintered porous materials. Figure 8b shows the value of elastic modulus by experimental and calculation. As seen in Figure 8b, the calculated data fit well with the value of the elastic modulus after nitriding and sintering (experimental data). However, the decrease in the calculated elastic modulus value is slightly lower than the experimental elastic modulus value with increasing porosity. This may be attributed to the less homogeneous distribution of pores compared with the theoretical distribution of pores [38] in the alloys with porosities of 14.3%, 26.3% and 37.8%.

## 4. Discussion

The elastic modulus of the as-sintered porous materials decreases with increasing amount of space holder, as shown in Figure 8. The relationship between the elastic modulus (E) and porosity (ε) of a porous material is given by the equation [38,39]:

(1) $$E=E_{o}[\frac{(1-\varepsilon {)}^{2}}{1+\varepsilon (2-3\gamma_{o})}]$$

where E_o_ is the elastic modulus of corresponding dense materials, with a value of 207 GPa; γ_o_ is the Poisson’s ratio of corresponding dense materials, with a constant of 0.305. The elastic modulus of the porous materials with different porosities obtained in this study was compared with the results calculated by the above equation, as shown in Table 2. According to the equations above, the elastic modulus of the porous materials with 0 wt. %, 10 wt. %, 20 wt. % and 30 wt. % space holder are calculated to be 131.8 GPa, 87.3 GPa, 56.7 GPa and 42.1 GPa, respectively, as shown in Table 2 and Figure 8b. It can be seen that the elastic modulus depends on the porosity. The elastic modulus decreases to approximately 40 GPa when the amount of space holder increases to 30 wt. %. Surprisingly, the sintered porous alloys exhibit a low elastic modulus of 40 GPa, which is close to the human cortical bone. This gradually decreasing property is mainly attributed to the porosity increase and density reduction. This kind of high-strength low-elastic-modulus high-N Ni-free austenitic stainless steel has the potential to be used as medical implant materials.

The as-fabricated porous HNASSs shows good yield strength and compressive strength, compared to common porous austenitic and ferritic stainless steel [1,2] or other porous metals [4,16]. The excellent mechanical properties are mainly ascribed to solid solution strengthening caused by N and precipitate strengthening caused by nitrides. The absorbed N exists in solid solution state or in nitrides. One study reported the relationship between yield strength (YS) and composition and microstructure of ASSs with the following equation [40,41]:

YS (MPa) = 63.5 + 496 N + 356.5 C + 20.1 Si + 3.7 Cr + 14.6 Mo + 18.6 V + 4.5 W + 40.3 Nb + 26.3 Ti + 12.7 Al + 2.5 δ + k^HP^d^−1/2^ (2)

where the element symbols denote the content of the elements (in wt. %), δ is the volume percent of ferrite, d is the average grain size of austenite matrix, and k^HP^ is the Hall-Petch coefficient. Solid solution N has the strongest effect in promoting yield strength of all of the elements. It can be seen that the effect factor of N is 1.4 times higher than that of C in the HNASSs. The superior mechanical properties of N-containing austenitic stainless steel become quite obvious. Simmons reported that nitrides had little effect on yield and ultimate strength, but reduced tensile ductility and impact toughness [18]. TEM results proved that both the worm-shaped and granular-shaped precipitate observed in Figure 3b is CrN phase. Large amounts of dislocations were tangled and concentrated on the edge of the nitrides, as shown in Figure 5a and Figure 6a. The CrN phase acts as obstacle for dislocation movement. Thus, it can be concluded that the CrN phase results in precipitate strengthening, which promotes yield strength.

The mechanical properties of porous HNASSs are tightly related with their porosity. A model was proposed to express the yield strength of porous materials, and an empirical equation was suggested by Gibson and Ashby [42]:

(3) $${\;\sigma }_{y}=\sigma_{\mathrm{ys}}C\rho_{\mathrm{rel}}^{3/2}(1+\rho_{\mathrm{rel}}^{2/1})$$

where σ_y_ denotes the yield strength of the porous materials, σ_ys_ refers to the yield strength of nonporous material, and ρ_rel_ is the relative density. C is a constant value of 0.23 for various bulk materials by Gibson and Ashby [42]:

(4) ρrel=ρρ0

where ρ is the density of the porous materials and ρ_0_ is the density of the nonporous materials. According to Equation (3), high-strength porous material can be produced by using high-strength dense material. HNASSs with excellent mechanical property are very suitable for fabricating high-strength porous material. Porous materials prepared by powder metallurgy can not only control the pores size and porosities, but can also adjust mechanical properties such as density, strength and elastic modulus of the material, so as to meet the requirement of specific application. The proposed powder metallurgical route made it possible to obtain porous HNASSs with predictable porosity, size, distributions of pores, elastic modulus and compressive strength, depending on the parameters of preparation (the amount of space holder and sintering temperature).

## 5. Conclusions

Porous high-N Ni-free austenitic stainless steels were successfully fabricated by gas-solid nitriding and sintering. The microstructure and mechanical properties of the porous alloys were investigated.

The addition of N into the duplex stainless steel promotes phase transformation from duplex stainless steel into austenitic stainless steel. A certain amount of worm-shaped and granular-shaped chromium nitrides were observed in the prepared high-N austenitic stainless steel. The nitrides were identified as face centered cubic CrN. The orientation relationship between CrN and the austenite matrix is [011]_CrN_//[011]_γ_ and (-1-11)_CrN_//(1-11)_γ_. Phase constituents of the as-received material and the sintered materials help to understand the relationship between the amount of nitrides and N content. The precipitate sequences of Cr nitrides in N-containing stainless steel are Cr_2_N, Cr_2_N + CrN, CrN, with increasing N content.

The 3D morphology of the sintered porous alloy shows superior pore connectivity, which makes this steel very beneficial for liquid transport and cell ingrowths for potential biocompatible materials. The pore size, distribution, and porosities, and thus the density, strength and elastic modulus, are controllable in this method.

Compressive strength and Young’s modulus all decreased with an increase in porosity. The elastic modulus decreases to approximately 40 GPa when the amount of space holder increases to 30 wt. %. The sintered porous materials exhibit a low elastic modulus of 40 GPa, and an ultra-large fracture strain of 20%, along with high yield strength of 360–380 MPa. Compared with other stainless steel foam or metal foam, the HNASS foam investigated in this research exhibits higher mechanical properties. These kinds of excellent mechanical properties are mainly ascribed to the solid solution strengthening caused by N and the precipitate strengthening caused by nitrides.

## Figures and Tables

**Table 1:** Sample codes and corresponding processing parameters.

Sample Codes		Detailed Processing Parameters		
		Sintering Temperature (°C)	Space Holder (wt. %)	Compressive Pressure (MPa)
A_1_	N1120-30-374	1120	30	374
A_2_	N1200-30-374	1200	30	374
A_3_	N1250-30-374	1250	30	374
A_4_	N1200-0-374	1200	0	374
A_5_	N1200-10-374	1200	10	374
A_6_	N1200-20-374	1200	20	374

**Table 2:** Summarized mechanical and physical properties of porous HNASSs.

Sample Codes		Porosity (%)	Compressive Strength (MPa)	Yield Strength (MPa)	Elastic Modulus (GPa)	Caculated Elastic Modulus (GPa)
A_1_	N1120-30-374	51.8	151.1	129.9	39.1 ± 7.0	30.8
A_2_	N1200-30-374	45.0	377.0	220.4	44.1 ± 5.0	42.1
A_3_	N1250-30-374	42.6	383.2	224.8	58.9 ± 2.9	46.7
A_4_	N1200-0-374	14.3	1759.8	767.6	161.9 ± 9.1	131.8
A_5_	N1200-10-374	26.3	1259.3	516.7	126.5 ± 8.2	87.3
A_6_	N1200-20-374	37.8	516.0	249.7	76.2 ± 6.7	56.7
